# KCTD1 stabilizes c-Myc to upregulate PD-L1 and suppress anti-tumor immunity in hepatocellular carcinoma

**DOI:** 10.1038/s41420-026-02975-6

**Published:** 2026-03-02

**Authors:** Dongmei Zhong, Shengwen Long, Yilan Dai, Yaru Yin, Zixin Zhang, Mi Ouyang, Xinyu Zhu, Anyi Hou, Yanling Qin, Qinghao Wang, Mengting Gong, Xiaofeng Ding

**Affiliations:** 1 The National & Local Joint Engineering Laboratory of Animal Peptide Drug Development, College of Life Science, Hunan Normal University, Changsha, China; 2 Institute of Interdisciplinary Studies, Hunan Normal University, Changsha, China; 3 College of Physical Education, Hunan University of Finance and Economics, Changsha, China

**Keywords:** Liver cancer, Immune evasion

## Abstract

Hepatocellular carcinoma (HCC) is the predominant histologic subtype of primary liver cancer and accounts for approximately 90% of cases worldwide. Although immune checkpoint blockade (ICB) therapies targeting the PD-1/PD-L1 axis have demonstrated clinical promise in advanced HCC, therapeutic responses remain heterogeneous, underscoring the need to elucidate the mechanisms governing PD-L1 expression. Here, we identify potassium channel tetramerization domain-containing protein 1 (KCTD1) as a previously unrecognized regulator of PD-L1 in HCC. Mechanistically, KCTD1 enhances PD-L1 expression through stabilizing of the oncoprotein c-Myc. Immunofluorescence and co-immunoprecipitation assays reveal a direct interaction between KCTD1 and c-Myc, mediated by the BTB domain of KCTD1 and the BR-HLH-LZ domain of c-Myc. Knockdown of KCTD1 leads to decreased c-Myc and PD-L1 protein levels, concomitant with increased production of pro-inflammatory cytokines, including IFN-γ and TNF-α, and augmented CD8⁺ T cell cytotoxic activity in vitro. In a murine intrahepatic tumor model, KCTD1 knockdown synergizes with anti–PD-1 therapy, resulting in enhanced tumor infiltration by CD4⁺ and CD8⁺ T lymphocytes and improved anti-tumor efficacy. These findings establish KCTD1 as a key modulator of immune evasion in HCC and a promising target to potentiate immune checkpoint therapy.

## Highlights

- KCTD1 interacts with c-Myc through its N-terminal BTB domain and the C-terminal BR-HLH-LZ domain of c-Myc.
- KCTD1 knockdown reduces the stability of the PD-L1 protein and enhances CD8^+^ T cell-mediated anti-tumor immunity.
- KCTD1 knockdown significantly improves the therapeutic efficacy of anti-PD-1 immune checkpoint blockade in HCC.

## Introduction

Hepatocellular carcinoma (HCC) is the most common form of primary liver cancer [1], and HCC originates primarily from the malignant transformation of hepatocytes, typically in the context of chronic liver injury, and represents a major global health burden due to its high incidence and mortality [2]. HCC patients are frequently diagnosed at an advanced stage when curative interventions such as surgical resection or liver transplantation are no longer feasible [3]. Despite recent therapeutic advances, the prognosis for advanced HCC remains poor, highlighting the need for more effective treatment strategies.

The advent of immune checkpoint blockade (ICB) has transformed the therapeutic landscape for several malignancies, including HCC. ICB therapies restore anti-tumor immunity by disrupting inhibitory receptor–ligand interactions between T cells and tumor cells, thereby reactivating cytotoxic immune responses [4]. Among the most clinically advanced ICB targets is the PD-1/PD-L1 axis, which has shown particular promise [5]. PD-1/PD-L1 blockade enhances T lymphocyte activation by inhibiting the interaction between PD-1 on lymphocytes and PD-L1 on tumor or stromal cells, thereby promoting anti-tumor immunity and avoiding immune evasion [6]. Monoclonal antibodies targeting PD-1 or PD-L1 have demonstrated efficacy in several cancers such as non-small cell lung cancer, breast cancer, and HCC [7]. However, response rates to PD-1/PD-L1 blockade in HCC remain modest, with objective responses observed in only ~16% of patients [8]. For example, the anti–PD-L1 antibody atezolizumab has shown clinical benefit in a subset of HCC patients, yet many fail to respond or eventually develop resistance [9]. Similarly, anti–PD-1 therapy with nivolumab has shown durable responses in a minority of advanced HCC cases, but adverse events and relapse remain concerns [10]. The overall efficacy of ICB remains limited in the majority of cancer patients, largely due to tumor-intrinsic and microenvironmental mechanisms that enable immune evasion under therapeutic pressure [11]. These limitations underscore the urgent need to elucidate the molecular mechanisms regulating PD-L1 expression and immune evasion in HCC, with the aim of identifying novel therapeutic targets and rational combination strategies.

The potassium channel tetramerization domain-containing (KCTD) protein family is characterized by a conserved N-terminal BTB (broad-complex, tramtrack, and bric-à-brac) domain and variable C-terminal regions, and has been implicated in a range of biological functions [12]. For instance, KCTD9 regulates natural killer (NK) cell maturation [13], and KCTD12 participates in neuronal signal transduction [14]. Despite emerging insights into individual KCTD family members, their roles in tumor immunity remain largely unexplored. KCTD1, a nuclear transcriptional repressor distinguished by a single BTB domain, has been shown to negatively regulate the transcription factor AP-2α, which in turn represses PD-L1 expression and promotes anti-tumor immune responses in glioma models [15, 16]. As PD-L1 upregulation is associated with immune evasion and poor prognosis in HCC [17], and given our recent discovery that KCTD1 promotes HCC progression by stabilizing HIF-1α and activating VEGF signaling [18], we hypothesized that KCTD1 may also modulate the PD-1/PD-L1 axis, potentially influencing the tumor immune microenvironment and disease progression in HCC.

In the present study, we identify KCTD1 as a novel upstream modulator of PD-L1 in HCC. We demonstrated that KCTD1 directly interacts with the oncoprotein c-Myc and enhances its stability, thereby promoting PD-L1 expression. Loss-of-function experiments reveal that KCTD1 knockdown reduces PD-L1 levels, enhances CD8⁺ T cell-mediated cytotoxicity, and increases the production of pro-inflammatory cytokines in vitro. Furthermore, KCTD1 knockdown augments the therapeutic efficacy of anti–PD-1 treatment in murine models of intrahepatic HCC. Collectively, these findings uncover an immunosuppressive role of KCTD1 and suggest that targeting the KCTD1–c-Myc–PD-L1 axis may improve the clinical outcomes of ICB in HCC.

## Results

### KCTD1 positively correlates with PD-L1 expression and predicts poor prognosis in HCC

**Fig. 1:**
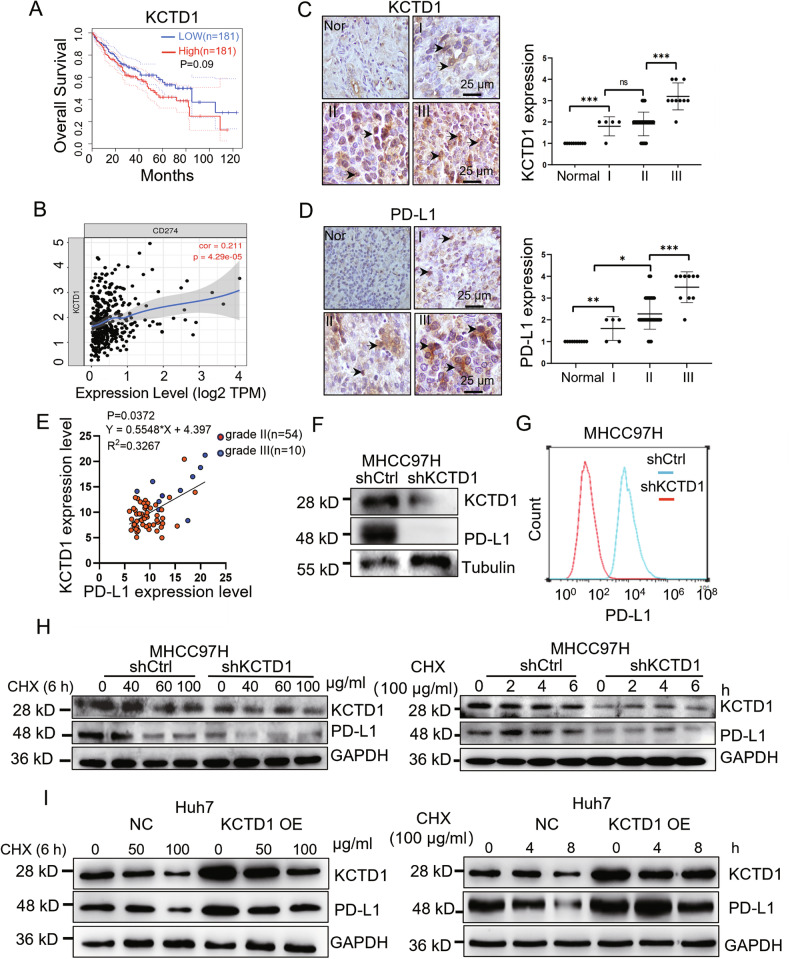
Positive correlation between KCTD1 and PD-L1 protein expression in HCC. **A**, **B** A database analyzed the survival of high/low expressed KCTD1 and the correlation between KCTD1 and PD-L1 expression in HCC. **C**, **D** IHC analysis of KCTD1 and PD-L1 expression and their scoring in HCC tissues with different grades. **E** Statistical analysis showed a positive correlation between KCTD1 and PD-L1 in HCC tissues, as determined by linear regression. **F**, **G** Western blots and FASC analysis of the correlation between the expression of KCTD1 and PD-L1 in HCC cells. **H**, **I** Western blots show that knocking down KCTD1 decreased while overexpressing KCTD1 enhanced PD-L1 protein stability in the CHX-treated cells.

Our recent studies have shown that KCTD1 promotes HCC proliferation and metastasis, and KCTD1 knockdown improves the therapeutic efficacy of sorafenib in HCC models [18]. Based on these findings, we investigated whether KCTD1 might modulate immune responses and influence the efficacy of ICB therapy in HCC. Programmed death-ligand 1 (PD-L1), a key immune checkpoint ligand, is predominantly expressed on tumor cells and mediates immune evasion [19]. we have discovered that HCC patients with low KCTD1 expression exhibit a longer survival time through the database (https://cistrome.shinyapps.io/timer/). More significantly, that KCTD1 expression is significantly upregulated in tumor tissues and positively correlates with PD-L1 expression (Fig. 1A, B). Immunohistochemistry (IHC) analysis further confirmed co-overexpression of KCTD1 and PD-L1 in high-grade HCC tissues compared with normal tissues (Fig. 1C–E). Knockdown of KCTD1 in MHCC97H cells markedly decreased PD-L1 expression, as shown by both Western blotting and flow cytometry (FACS) (Fig. 1F,G). These results indicate a positive correlation between KCTD1 and PD-L1 expression in HCC. Cycloheximide (CHX) chase assays demonstrated that KCTD1 knockdown decreased PD-L1 protein stability at the indicated timepoints or at different CHX concentrations compared with the control groups (Fig. 1H). Conversely, the overexpression of KCTD1 led to a higher stability of the PD-L1 protein (Fig. 1I), indicating that KCTD1 stabilizes PD-L1 at the post-translational level. Together, these results suggest that KCTD1 facilitates immune evasion in HCC by stabilizing PD-L1, highlighting its potential as a target to enhance the immunotherapy efficacy.

### KCTD1 regulates PD-L1 expression through c-Myc

**Fig. 2:**
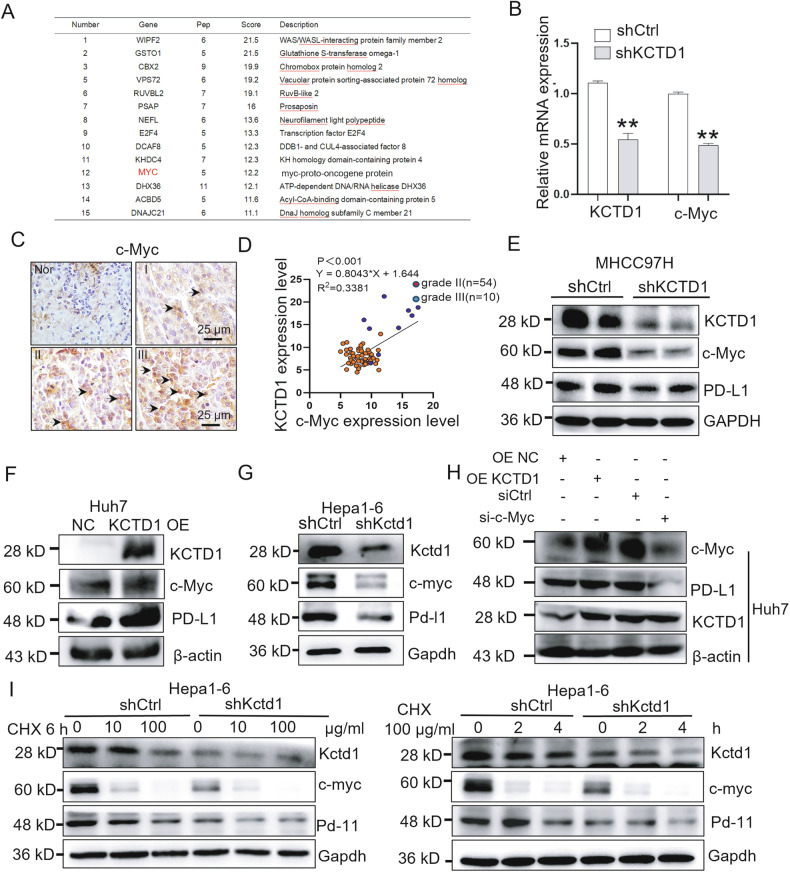
KCTD1 regulates PD-L1 expression via c-Myc in HCC. **A** silver staining and mass spectrometry identifying KCTD1-associated proteins in MHCC97H cells. **B** qRT-PCR analysis of *KCTD1* and *c-Myc* mRNA levels in HCC tissues. **C**, **D** Representative IHC images and quantification of KCTD1 and c-Myc expression correlation in HCC tissues. **E**–**G** Western blot validation of KCTD1–c-Myc expression correlation in various HCC cell lines with KCTD1 knockdown or overexpression. **H** PD-L1 expression following c-Myc knockdown in KCTD1-overexpressing HCC cell lines. **I** c-Myc protein degradation in KCTD1-knockdown cells under varying CHX concentrations and time points.

To uncover the molecular mechanism by which KCTD1 regulates PD-L1, we performed co-IP assays using KCTD1-specific antibodies in MHCC97H cells, followed by silver staining of the immunoprecipitated proteins and mass spectrometry analysis of the resulting bands (Supplementary Fig. 1). Among the top 15 candidate interacting proteins (Fig. 2A), c-Myc, a well-established transcription factor known to regulate PD-L1 and modulate tumor immune responses, was identified as a candidate protein that interacts with KCTD1 [20]. Real-time Quantitative Reverse Transcription PCR (qRT-PCR) analysis revealed that KCTD1 knockdown significantly reduced c-Myc transcript levels (Fig. 2B), and IHC analysis showed high c-Myc expression in high-grade HCC and a positive correlation between KCTD1 and c-Myc expression was found in grade II/III HCC tissues (Fig. 2C, D). Western blot analyzes further demonstrated that the expression levels of c-Myc and PD-L1 proteins were positively correlated with KCTD1 expression in HCC cell lines, decreasing upon KCTD1 knockdown and increasing upon its overexpression.(Fig. 2E–G), whereas knockdown of c-Myc alone decreased PD-L1 expression without affecting KCTD1 levels in Huh7 cells (Fig. 2H), indicating that c-Myc functions downstream of KCTD1 and both contribute to the regulation of PD-L1 expression. CHX chase assays confirmed that knockdown of KCTD1 decreased the protein stability of both c-Myc and PD-L1, whereas KCTD1 overexpression increased their stability (Fig. 2I). Collectively, these results identify a KCTD1–c-Myc axis that regulates PD-L1 expression in HCC.

### KCTD1 interacts with c-Myc via the BTB and BR-HLH-LZ domains to modulate PD-L1 expression

**Fig. 3:**
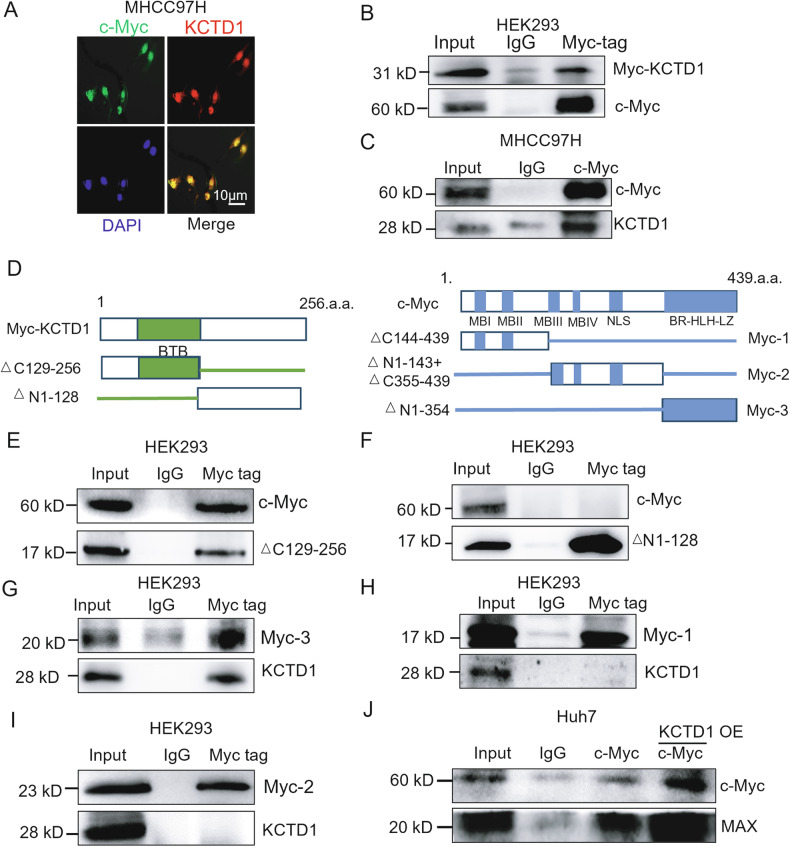
KCTD1 directly interacts with c-Myc. **A** Immunofluorescence imaging showing nuclear colocalization of KCTD1 and c-Myc proteins in HCC cells. **B**, **C** co-IP analysis using both exogenous and endogenous proteins confirms the physical interaction between full-length KCTD1 and c-Myc. **D** Schematic representation of the domain structures of KCTD1 and c-Myc proteins. **E**, **F** co-IP analysis of the interaction between truncated KCTD1 and endogenous c-Myc. **G**–**I** co-IP analysis of the interaction between truncated c-Myc and endogenous KCTD1, revealing the BTB domain of KCTD1 mediates the interaction with the BR-HLH-LZ domain of c-Myc. **J** co-IP assay showing the effect of KCTD1 overexpression on the interaction between c-Myc and its partner MAX in Huh7 cells.

KCTD1 is a nuclear protein known to primarily function via the conserved N-terminal BTB domain [10], whereas *c-Myc* is synthesized in the cytoplasm and exerts its transcriptional activity in the nucleus through heterodimerization with MAX [21]. Immunofluorescence analysis revealed nuclear co-localization of KCTD1 and c-Myc in MHCC97H cells (Fig. 3A). HEK293 cells were transiently transfected with pCMV-Myc-KCTD1 expression plasmids. Co-immunoprecipitation using an anti-Myc-tag antibody demonstrated that endogenous c-Myc was co-immunoprecipitated with Myc-KCTD1 (Fig. 3B). Consistently, a physical association between endogenous KCTD1 and c-Myc proteins was also confirmed (Fig. 3C). To map the interaction domains, we generated truncated constructs of both proteins (Fig. 3D). Subsequent co-IP analysis using these constructs revealed that only the Δ129-256 fragment of KCTD1 containing the BTB domain was able to form a complex with endogenous c-Myc proteins (Fig. 3E, F) while the Δ1-354 fragment of c-Myc with the BR-HLH-LZ domain mediated this interaction with endogenous KCTD1 proteins (Fig. 3G–I). These results indicate that the BTB domain of KCTD1 directly interacts with the BR-HLH-LZ domain of c-Myc. We next examined the functional consequences of this interaction. Overexpression of KCTD1 in Huh7 cells promoted the formation of c-Myc–MAX heterodimers and enhanced c-Myc protein stability (Fig. 3J). These findings establish that KCTD1 interacts with c-Myc in the nucleus to promoter PD-L1 expression, identifying the KCTD1–c-Myc–PD-L1 axis as a potential therapeutic target in HCC.

### KCTD1 knockdown enhances CD8^+^ T cell-mediated anti-tumor immunity in vitro

**Fig. 4:**
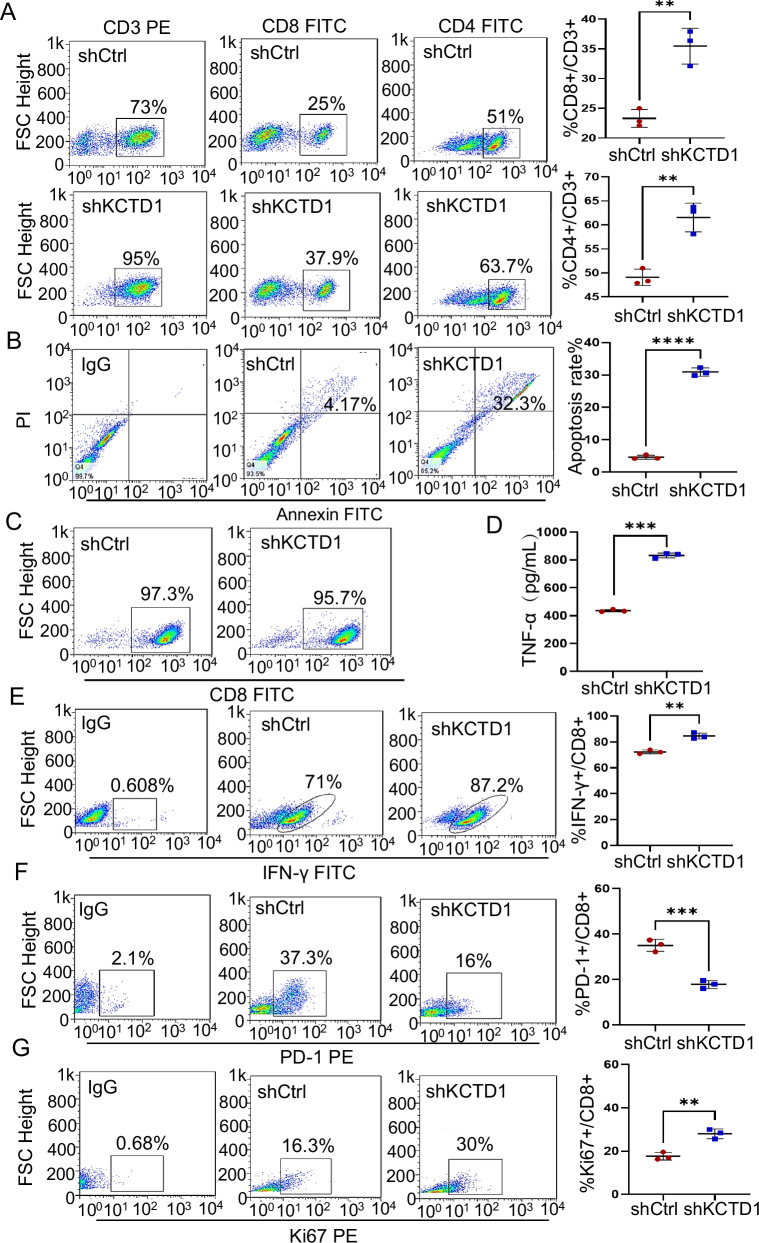
KCTD1 knockdown enhances CD8^+^ T cell-mediated anti-tumor responses in vitro. **A** FACS analysis of CD8⁺/CD3⁺and CD4⁺ /CD3⁺ T cell proportions in PBMCs culture with MHCC97H cells. **B** Quantification ratio of apoptotic tumor cells after co-culture with PBMCs. **C** FACS analysis of CD8⁺ T cell purity following isolation from the PBMC-MHCC97H co-culture system. **D** ELISA quantification of TNF-α secretion in culture supernatants. **E**–**G** FACS analysis of IFN-γ production, PD-1 expression, and Ki67 levels in CD8⁺ T cells following co-culture.

Engagement of PD-L1 on tumor cells with PD-1 on CD8^+^ T cells facilitates tumor immune evasion [22]. To assess the immunomodulatory role of KCTD1, we co-cultured human peripheral blood mononuclear cells (PBMCs) with MHCC97H cells. KCTD1 knockdown in tumor cells significantly increased the proportions of CD4^+^/CD3^+^ and CD8^+^/CD3^+^ T cell subsets (Fig. 4A). The apoptosis of MHCC97H cells was both enhanced compared with the controls when cocultured with PBMCs (Fig. 4B), indicating increased T cell–mediated cytotoxicity. To specifically examine the anti-tumor effect of KCTD1 knockdown on CD8^+^ T cells, 97.3% and 95.7% purity of CD8^+^ T cells from co-culture systems were enriched and specifically evaluated (Fig. 4C). Enzyme-Linked Immunosorbent Assay (ELISA) analysis demonstrated the increased secretion of TNF-α, and FACS analysis revealed elevated production of IFN-γ by CD8^+^ T cells upon KCTD1 knockdown (Fig. 4D, E), indicating enhanced cytotoxic function. Additionally, decreased PD-1 levels and increased Ki-67 expression in CD8^+^ T cells showed augmented proliferation and activation (Fig. 4F, G, and Supplementary Fig. 2) [23, 24]. Together, these data demonstrate that silencing KCTD1 in HCC cells augments CD8^+^ T cell-mediated cytotoxicity and anti-tumor immune responses.

### KCTD1 promotes HCC growth and immune evasion in vivo

**Fig. 5:**
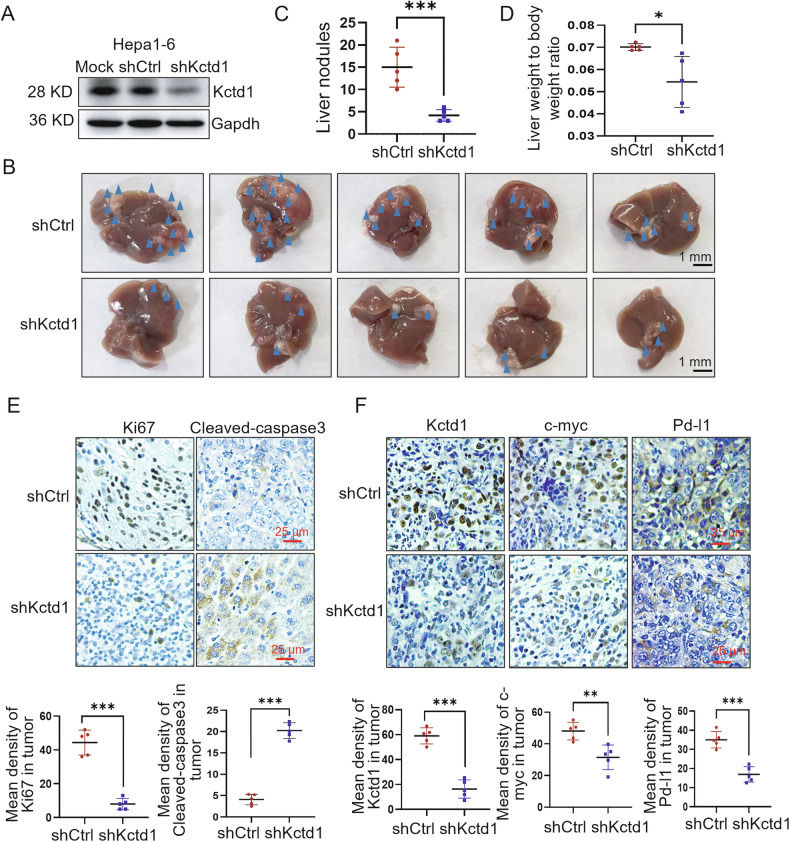
Kctd1 knockdown inhibits tumor growth and enhances immunogenicity in a murine HCC model. **A** Western blots confirmation of Kctd1 knockdown in Hepa1-6 cells. **B**, **C** Representative images and quantification of intrahepatic tumor nodules in mice injected with control or Kctd1-knocked down cells. **D** Analysis of liver-to-body weight ratios. **E**, **F** IHC analysis and quantification of Ki67, cleaved-caspase3, Kctd1, c-myc and Pd-l1 expression in liver tumors from each experimental group.

To evaluate the role of KCTD1 in tumor progression in vivo, we established an orthotopic HCC model by implanting Kctd1-silenced Hepa1-6 cells into the livers of C57BL/6 mice. Efficient knockdown of Kctd1 was confirmed (Fig. 5A, and Supplementary Fig. 3). On day 15 post implantation, knockdown of Kctd1 significantly reduced hepatic tumor nodules and decreased the liver-to-body weight ratio (Fig. 5B–D). Moreover, IHC analysis revealed lower Ki-67 expression and higher cleaved-caspase 3 expression in Kctd1-knockdown tumor tissues, indicating reduced proliferative and increased apoptosis compared to control tissues (Fig. 5E). And the expression of c-Myc and Pd-l1 was consistently reduced in Kctd1-knockdown tumors (Fig. 5F). These results support the functional role of KCTD1 in promoting HCC progression through the c-Myc/PD-L1 axis.

To determine the involvement of CD8^+^ T cells in the regulation of tumor progression by KCTD1, anti-Cd8a antibodies were administered intraperitoneally prior to and during tumor development (Supplementary Fig. 4A). Depletion of CD8^+^ T cells abrogated the tumor-suppressive effect of Kctd1 knockdown, compared with the control group, and tumor progression was more pronounced, reflected by an increased liver-to-body weight ratio and a higher number of liver nodules (Supplementary Fig. 4B–D). IHC results confirmed that the expression of Cd8 in the spleen of mice injected with the anti-Cd8a antibody was significantly reduced (Supplementary Fig. 4E, F). These findings demonstrated that KCTD1 regulates tumor growth, at least in part, through modulation of CD8⁺ T cell-dependent immunity.

### KCTD1 knockdown potentiates the efficacy of anti-PD-1 immunotherapy

**Fig. 6:**
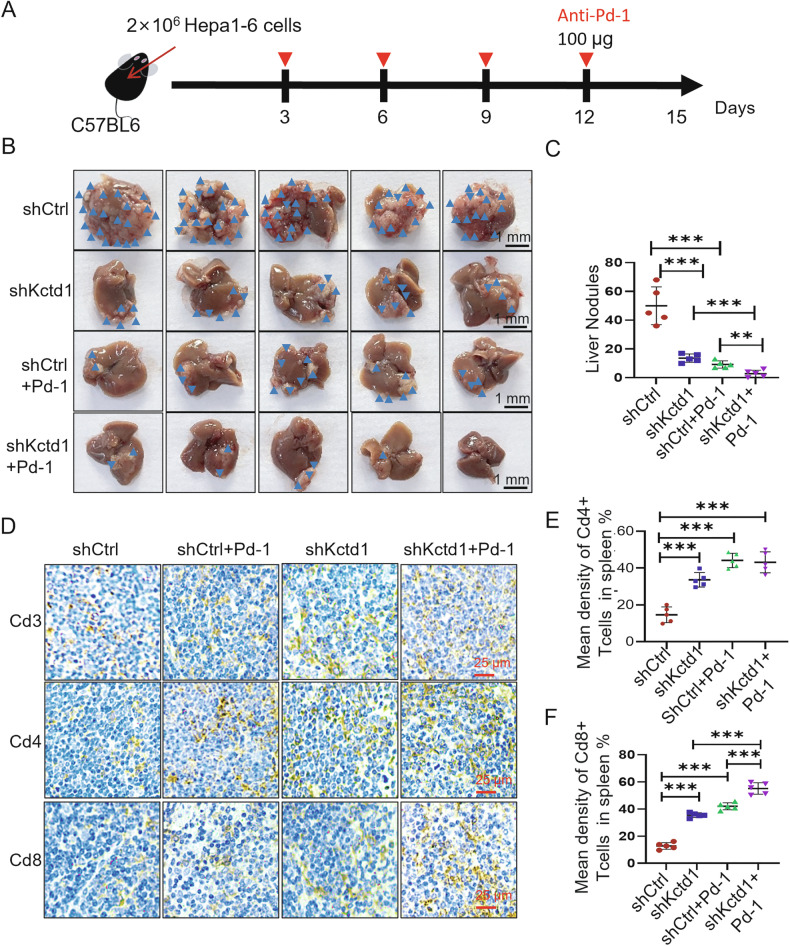
Kctd1 knockdown synergizes with anti–PD-1 therapy to suppress tumor progression in vivo. **A** Schematic diagram of the treatment strategy for intrahepatic HCC tumors in C57BL/6 mice. **B** Representative images of intrahepatic livers following Kctd1 knockdown and/or anti–PD-1 antibody treatment. **C** Quantification of tumor burden, including the number of tumor nodules. **D**–**F** IHC analysis of Cd3⁺, Cd4⁺, and Cd8⁺ T cell infiltration in the spleens from four treatment groups, along with quantification of positively stained cells.

To explore the therapeutic implications of targeting KCTD1, we tested whether its knockdown could enhance anti–PD-1 efficacy. C57BL/6 mice bearing orthotopic Kctd1-deficient Hepa1-6 tumors were treated with anti–PD-1 antibodies every 3 days beginning on day 3 post implantation (Fig. 6A). After 15 days of treatment, the average body weight of mice remained largely unchanged across groups, indicating minimal systemic toxicity (Supplementary Fig. 5, and Supplementary Table 2). Tumor imaging revealed a marked reduction in hepatic tumor nodules in the Kctd1 knockdown group compared to NC group. Notably, the combination of Kctd1 knockdown and anti–PD-1 therapy resulted in the most pronounced tumor regression with fewer liver nodules (Fig. 6B, C). IHC analysis of tumor-infiltrating lymphocytes revealed increased frequencies of CD3^+^, CD4^+^, and CD8a^+^ T cells in the Kctd1-knockdown tumors (Fig. 6D, E), and CD8a^+^ T cells were further augmented by combination treatment with anti-PD-1 antibodies (Fig. 6F). These findings demonstrate that KCTD1 knockdown not only promotes anti-tumor immunity but also synergizes with ICB therapy, providing a rationale for targeting KCTD1 in combination with PD-1 inhibitors in HCC.

## Discussion

The KCTD protein family has been implicated in diverse biological processes, including tumorigenesis [25]. Among them, KCTD1 is unique in that, unlike other family members such as KCTD5, which primarily function as E3 ubiquitin ligase adaptors [26], KCTD1 facilitates protein-protein interactions and modulates various signaling pathways [27]. Notably, KCTD1 has been shown to interact with KCASH1 and KCASH2, leading to reduced HDAC1 protein levels, acetylation-mediated inactivation of Gli1, and suppression of Hedgehog (Hh) signaling–dependent tumor cell proliferation [28]. More recently, KCTD1 has been identified as selectively expressed in T-cell acute lymphoblastic leukemia (T-ALL), underscoring its potential as a diagnostic biomarker in hematologic malignancies [29]. Building on these findings, our study reveals a previously unrecognized role of KCTD1 in modulating immune escape in HCC. Specifically, we identified KCTD1 as a novel regulator of PD-L1 expression and demonstrate its functional significance in modulating the tumor immune microenvironment both in vitro and in vivo.

PD-L1 is frequently overexpressed in tumor cells and mediates immune evasion by engaging PD-1 on T cells, thereby attenuating T cell–mediated cytotoxicity [30]. ICB targeting the PD-1/PD-L1 axis has transformed the landscape of cancer therapy [31]. Through integrated bioinformatic analysis of the cancer genome atlas (TCGA) and immunohistochemical staining of clinical HCC samples, we identified a positive correlation between KCTD1 and PD-L1 expression, highlighting a potential immunomodulatory function for KCTD1. Mechanistically, knockdown of KCTD1 reduced PD-L1 protein stability in the presence of CHX, suggesting that KCTD1 regulates PD-L1 at the post-translational level. These findings position KCTD1 as a potential biomarker and therapeutic target in the context of PD-1/PD-L1–based immunotherapy.

**Fig. 7:**
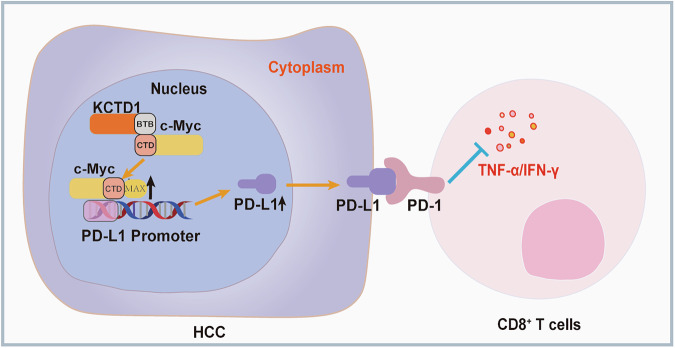
Mechanistic model of KCTD1 in regulating anti-tumor immunity in hepatocellular carcinoma. KCTD1 interacts with c-Myc through its BTB domain and C-terminal BR-HLH-LZ domain, facilitating the formation and stabilization of the c-Myc–MAX dimer, thereby promoting the transcriptional activation of PD-L1, leading to increased PD-L1 protein expression. Elevated PD-L1 suppresses CD8⁺ T cell–mediated cytotoxicity, thereby promoting immune evasion and tumor progression in hepatocellular carcinoma.

Further mechanistic insights revealed that KCTD1 regulates PD-L1 expression through stabilization of the proto-oncogene c-Myc. As a central transcriptional hub, c-Myc orchestrates key aspects of tumor biology, including proliferation [32], metabolic reprogramming [33], angiogenesis [34], and immune modulation [35]. c-Myc also contributes to immune evasion by regulating the expression of checkpoint molecules such as PD-L1 and CD47, and by altering the function of effector immune cells, including CD8⁺ T cells, NK cells, and tumor-associated macrophages [36]. Inhibition of c-Myc has been shown to enhance IFN-γ production by CD8⁺ T cells and to suppress tumor burden [37]. In our study, KCTD1, c-Myc, and PD-L1 exhibited coordinated expression patterns in HCC cell lines. We further demonstrated that KCTD1 physically interacts with c-Myc via its BTB domain, enhancing c-Myc protein stability. The interaction, in turn, promotes PD-L1 transcriptional activity and upregulation (Fig. 7). These findings reveal a previously uncharacterized KCTD1–c-Myc–PD-L1 signaling axis that drives immune evasion in HCC. Given the central role of BTB domains in mediating protein–protein interactions [38], our data suggest that the BTB domain of KCTD1 may represent a promising therapeutic target. Small-molecule inhibitors targeting BTB domains, such as BCL6-IN-9, which is under investigation for diffuse large B-cell lymphoma, exemplify the drug ability of this protein family [39]. Our findings suggest that the pharmacological disruption of the KCTD1–c-Myc interaction could destabilize PD-L1 and enhance the efficacy of anti–PD-1 therapy in HCC.

HCC is characterized by an immunosuppressive tumor microenvironment and limited infiltration of effector lymphocytes, which constrain the efficacy of immunotherapies [2]. Among immune effector cells, CD8⁺ T cells are key mediators of anti-tumor immunity and response to ICB [40]. Previous studies have shown that blockade of PD-1 signaling can restore CD8⁺ T cell function and impede tumor growth, whereas depletion of CD8⁺ T lymphocytes accelerates HCC progression [41–43]. Consistent with these observations, our data show that tumors with low KCTD1 expression exhibited increased infiltration of CD8⁺ T cells. In co-culture assays with PBMCs, knockdown of KCTD1 in HCC cells significantly impaired tumor cell viability and enhanced CD8⁺ T cell activation, as indicated by elevated expression of Ki67 and IFN-γ and reduced PD-1 levels. These results demonstrate that KCTD1 regulates both tumor-intrinsic immune evasion via PD-L1 and tumor-extrinsic immune suppression by modulating T cell effector function.

## Conclusion

In summary, our findings establish KCTD1 as a novel modulator of immune evasion in HCC. Mechanistically, KCTD1 stabilizes and activates c-Myc via a direct interaction through its BTB domain, resulting in increased PD-L1 expression and suppression of CD8⁺ T cell–mediated immunity. Functional validation using co-culture systems and murine models confirmed the immunosuppressive role of KCTD1 in HCC. Importantly, KCTD1 knockdown synergizes with anti–PD-1 treatment, providing a rationale for targeting KCTD1 or its interaction with c-Myc as a novel immunotherapeutic strategy to overcome resistance to ICB in HCC.

## Methods

### Immunohistochemistry (IHC) analysis

All experiments involving human tissues were approved by the Ethics Committee of Hunan Normal University, and informed consent was obtained from all participants. A total of 79 formalin-fixed paraffin-embedded liver tissue samples were used, including 10 normal controls, 5 grade I, 54 grade II, and 10 grade III HCC specimens. IHC was performed as previously described [44]. Primary antibodies included anti-KCTD1 (bs-16924R, Bioss, Beijing, China; 1:200), anti-PD-L1 (66248-1, Proteintech, Chicago, USA; 1:500), anti-c-Myc (10828-1-ap, Proteintech; 1:200), anti-CD3 (sc-20047, Santa Cruz, California, USA; 1:100), anti-CD4 (MT310, Santa Cruz; 1:100), anti-CD8a (70306, Cell Signaling, Boston, USA; 1:200) or IgG isotype control (ab37355, Abcam, Britain; 1:500).

### Cell culture and transfection

Authenticated HCC cell lines MHCC97H, Huh7, Hepa1-6 (American Type Culture Collection) were cultured in Dulbecco’s modified Eagle medium (DMEM, Gibco BRL, Grand Island, NY, USA) with 10% fetal calf serum and penicillin-streptomycin solution. PBMCs were cultured in complete RPMI 1640 medium (Gibco). Cells were maintained at 37 °C in a humidified incubator with 5% CO₂. Plasmid and siRNA transfections were performed using JetPrime reagent (Polyplus) following the manufacturer’s instructions [45].

### Lentiviral-mediated KCTD1 Knockdown

Stable knockdown of human or murine KCTD1 was achieved using lentiviral vectors (GenePharma, Athens). The KCTD1-targeting shRNA sequences were cloned into the GV248 vector (Genepharma, Shanghai, China). Cells were infected at a multiplicity of infection (MOI) of 10 in the presence of polybrene (REVGC03-1, GENE). Infected cells were selected with puromycin and visualized under a fluorescence microscope (Axio Imager M2, ZEISS, Germany) [18].

### PBMC isolation and CD8^+^ T cell sorting

PBMCs were isolated from healthy donors by the Ficoll method (P8610, Solarbio, Beijing, China). Briefly, peripheral blood was mixed with an equal volume of Ficoll and centrifuged at 600 × *g* for 20 min. The mononuclear cell layer was harvested, subjected to RBC lysis, washed and activated with 5 µM phytohemagglutinin and 75 IU/mL IL-2 for 72 h [16]. CD8⁺ T cells were magnetically separated using CD8 MicroBeads (130-045201, Miltenyi Biotec, Bergisch Gladbach, Germany).

### Flow cytometry

HCC cells and PBMCs were co-cultured at a 1:1 effector-to-target ratio for 12–16 h. Apoptosis was assessed using Annexin V-FITC/PI staining (Beyotime, Shanghai, China) [16]. For immunophenotyping, CD8^+^ T cells were fixed, blocked, and stained with fluorochrome-conjugated antibodies, including CD3-PE (PE-65133, Thermo Fisher Scientific, USA), CD4-FITC (FITC-65143, Thermo Fisher Scientific) and CD8-FITC (FITC-65144, Proteintech), PD-1-PE (11-9969-41, Thermo Fisher Scientific), anti-IFN-γ-FITC (11-7311-82, eBioscience, California, USA), and Ki67-PE (350053, BioLegend, California, USA). For intracellular Ki67 detection, cells were fixed, permeabilized using the Cyto-Fast™ Fix/Perm Buffer (426803, BioLegend), and stained. Data acquisition was performed using a FACSCalibur flow cytometer (BD, New Jersey, USA), and data were analyzed with FlowJo software.

### ELISA

TNF-α levels in culture supernatants were measured using a Human TNF-α ELISA MAX Deluxe kit (430204, BioLegend) [17]. Briefly, plates were coated with capture antibodies overnight, followed by incubation with samples or standards for 2 h. Human TNF-α ELISA MAX™ Detection Antibodies, HRP-conjugate, TMB substrate, and stop solution were then added sequentially, and absorbance was measured at 450 nm.

### Western blot

Cells were lysed in RIPA buffer (Bioss, C-0013), denatured at 105 °C, and subjected to SDS-PAGE [46]. Proteins were transferred to PVDF membranes and probed with antibodies against PD-L1, GAPDH (A01020, Abbkine, Wuhan, China), KCTD1, c-Myc, and β-actin (AP0063, Abbkine). Blots were visualized using an automatic chemiluminescence system (TACAN, USA). Original uncropped blot scans are provided in the Supplementary information.

### Plasmid construction

Full-length and truncated human c-Myc fragments including N-terminal (1–143 aa), central (144–354 aa), and C-terminal (355–439 aa) were PCR-amplified and cloned into the pCMV-Myc vector (Clontech). Full-length and truncated human KCTD1 recombinant plasmids were generated as previously described [15, 38]. The specific primer sequences were listed in Supplementary Table 1. All constructs were verified by Sanger sequencing (Sangon Biotech).

### Immunofluorescence colocalization

MHCC97H cells were seeded onto glass coverslips and cultured to about 40% confluence. Cells were treated as previously described [47]. Cells were fixed and stained sequentially with rabbit anti-KCTD1 (1:500), HRP-conjugated secondary antibodies and CY3-TSA (AS008, Servicebio), followed by rabbit anti–c-Myc (1:1000), HRP-conjugated secondary antibodies and FITC-TSA amplification. Nuclei were counterstained with DAPI (G1012, Servicebio) and images were acquired with the fluorescence microscope.

### Co-immunoprecipitation

HCC cells grown to ~80% confluence in 10-cm dishes were transfected with Myc-tagged full-length or truncated KCTD1 or c-Myc truncated plasmids. After 48 h, cells were lysed in RIPA buffer supplemented with protease inhibitors [47]. Lysates were pre-cleared and immunoprecipitated with rabbit anti-Myc antibody and Protein A/G PLUS agarose beads (P2108-1, BeyoMag). Immunoprecipitates were resolved by SDS-PAGE and probed with anti–c-Myc, anti-KCTD1, and anti-Myc antibodies. Rabbit IgG (550124, Zenbio) was used negative control.

### Murine intrahepatic HCC model and in vivo antibody blockade

The ethics committee from Hunan Normal University approved all mouse experiments, all experimental results met the standard of animal welfare and ethical. Four-week-old female C57BL/6J mice (Olex, Changsha, China) were randomly divided into 2 groups, with 5 mice in each group for orthotopically injected intrahepatically with 2 × 10⁶ Hepa1-6 cells in 35 μL PBS [46]. For in vivo antibody treatments, mice received intraperitoneal injections of 100 μg of anti-CD8 antibody (YTS169.4, BioXCell) on days –1, 0, and every 3 days and/or anti–PD-1 antibody (bsm-43072M, Bioss) on days 3, 6, 9, and 12. Mice were sacrificed on day 15 for analysis of liver/spleen weight and tumor burden. Tumor tissues were processed for IHC.

### Statistical analysis

All results are presented as mean ± SD from at least three independent experiments. Data were analyzed using ImageJ and GraphPad Prism (GraphPad Software, San Diego, CA). Statistical significance was evaluated using two-tailed unpaired Student’s *t*-tests or one-way analysis of variance as appropriate. Survival data were analyzed by Kaplan–Meier plots. *P*-values < 0.05 were considered statistically significant.

## Supplementary information

supplement results

supplemental information

original uncropped

## Data Availability

All data generated or analyzed during this study are available from the corresponding author on reasonable request.
