# Supplementary material for: KCTD1 stabilizes c-Myc to upregulate PD-L1 and suppress anti-tumor immunity in hepatocellular carcinoma
Source: Cell Death Discov. 2026 Mar 2;12:129. doi: 10.1038/s41420-026-02975-6 (PMC13039683; doi:10.1038/s41420-026-02975-6)
Supplement: Supplementary file 1 — supplement results [file 41420_2026_2975_MOESM1_ESM.pptx]

## Slide 1
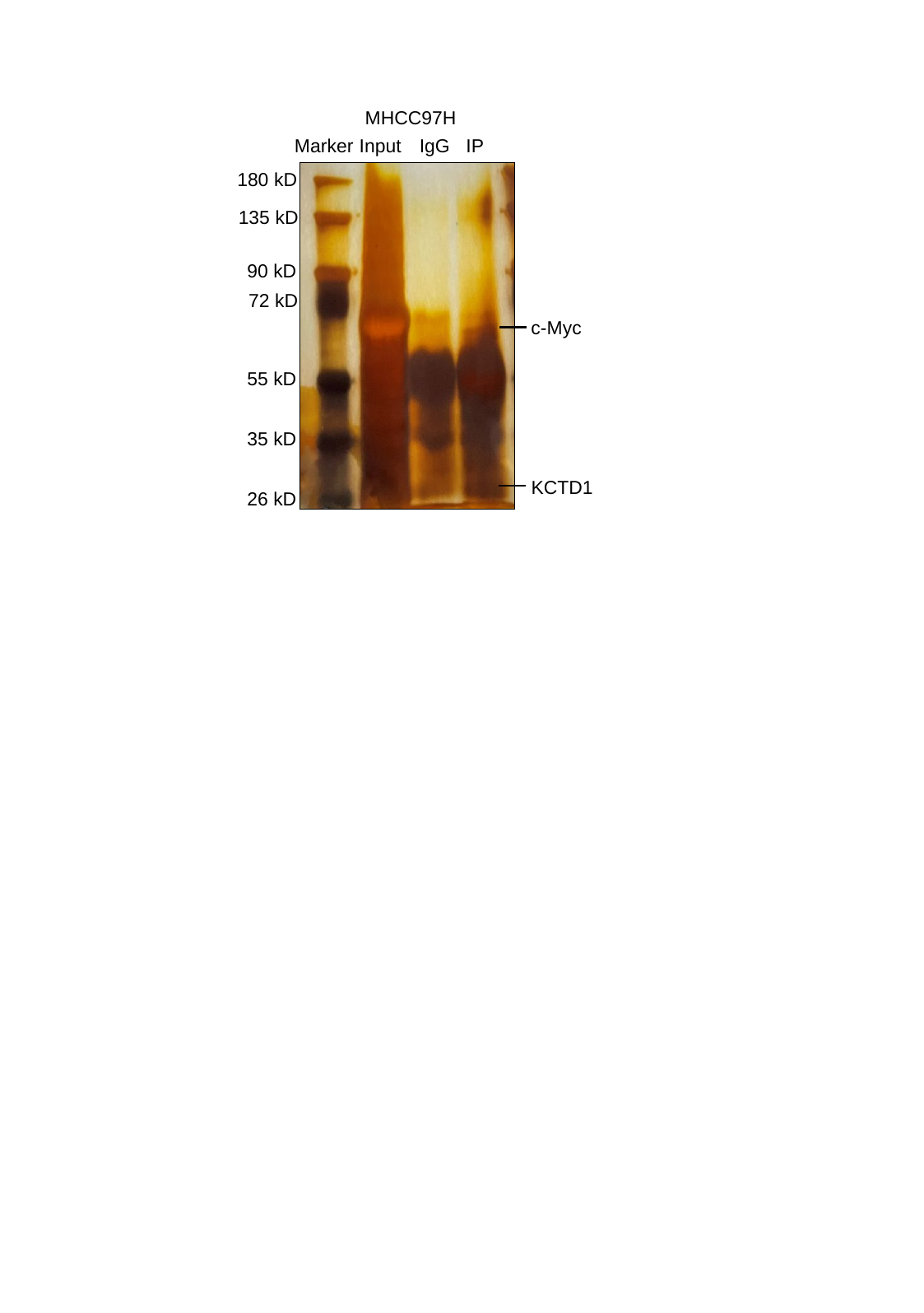

MHCC97H
Marker
Input
IgG IP
180 kD
135 kD
90 kD
72 kD
c-Myc
55 kD
35 kD
KCTD1
26 kD

## Slide 2
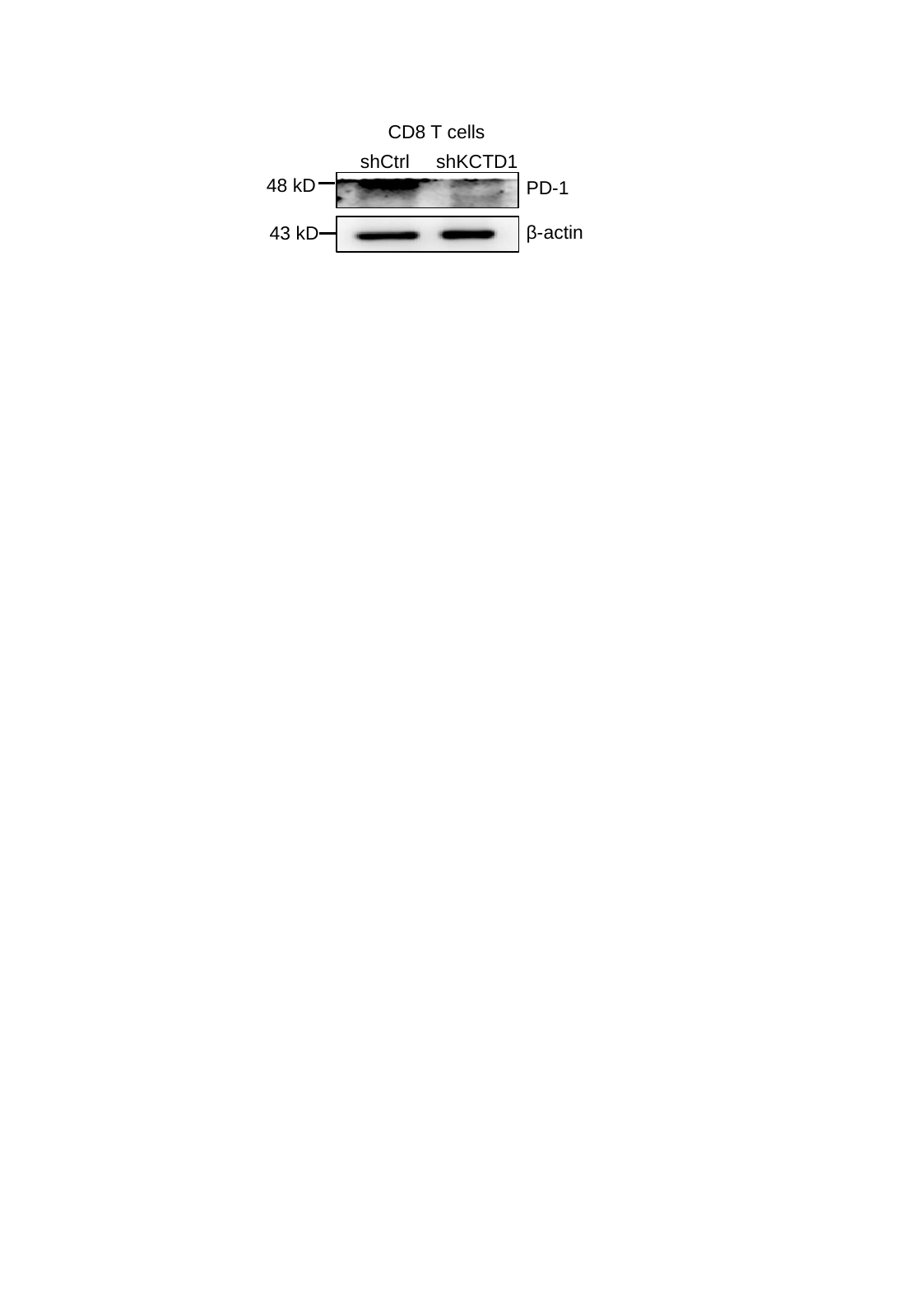

CD8 T cells
 shCtrl shKCTD1
48 kD
PD-1
43 kD
β-actin

## Slide 3
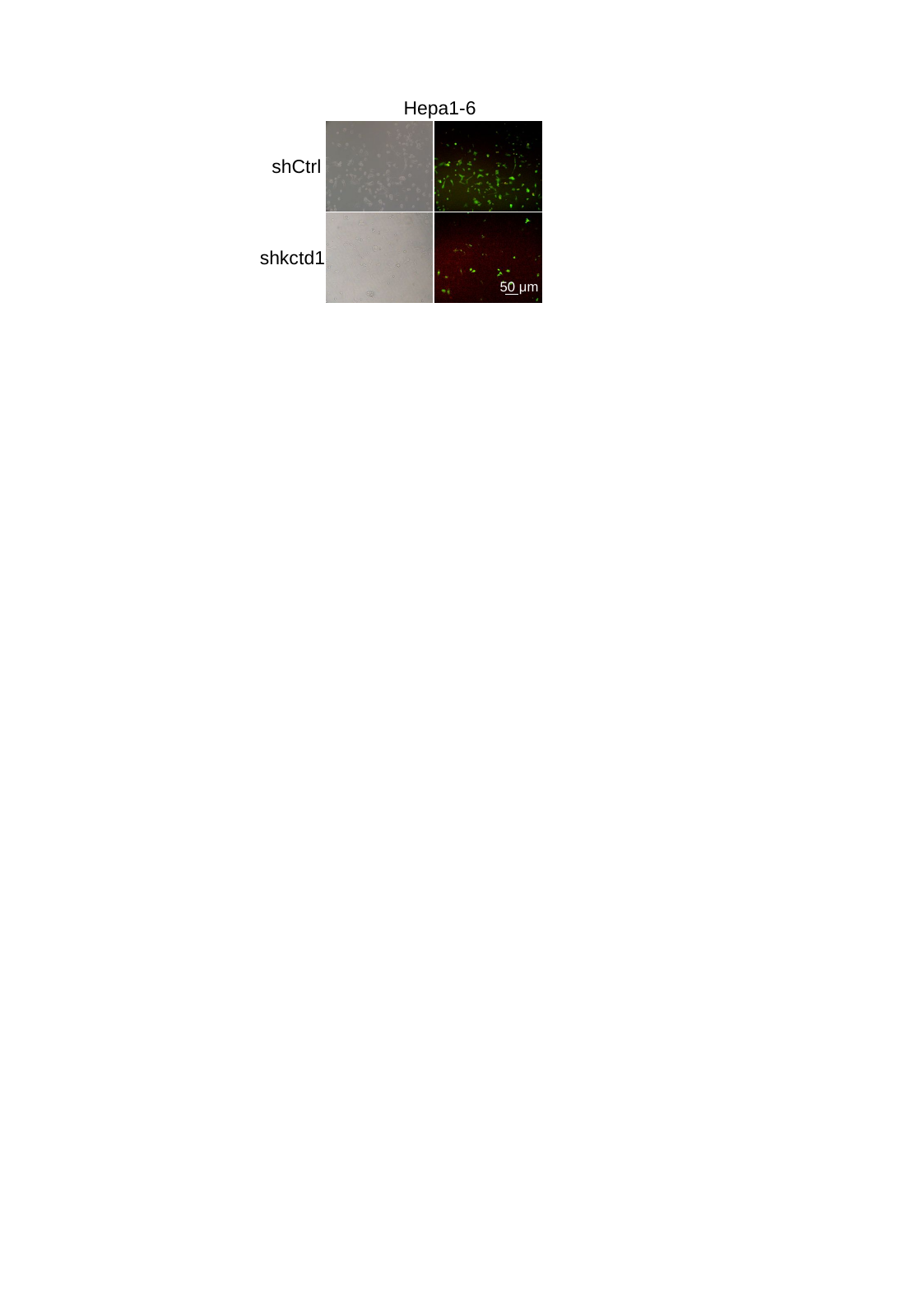

Hepa1-6
50 μm
shCtrl
shkctd1

## Slide 4
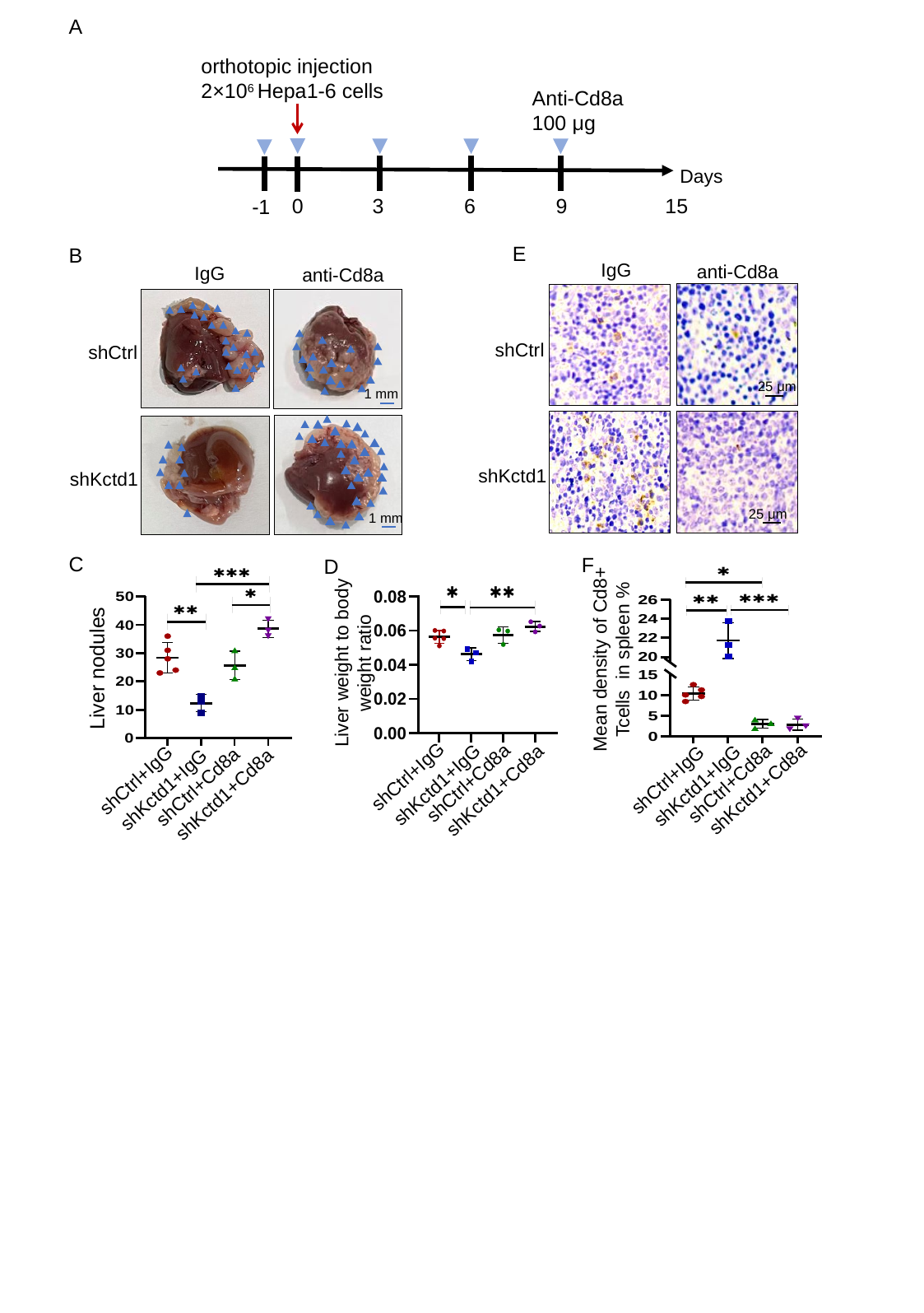

A
orthotopic injection
2×10^6^ Hepa1-6 cells
Anti-Cd8a
100 μg
Days
 0 3 6 9 15
-1
E
B
IgG
shCtrl
shKctd1
anti-Cd8a
1 mm
1 mm
IgG
anti-Cd8a
shCtrl
25 μm
shKctd1
25 μm
shCtrl+IgG
shCtrl+Cd8a
shKctd1+IgG
shKctd1+Cd8a
Liver nodules
Mean density of Cd8+ Tcells in spleen %
shCtrl+IgG
shCtrl+Cd8a
shKctd1+IgG
shKctd1+Cd8a
C
F
D
shCtrl+IgG
shCtrl+Cd8a
shKctd1+IgG
shKctd1+Cd8a
Liver weight to body weight ratio

## Slide 5
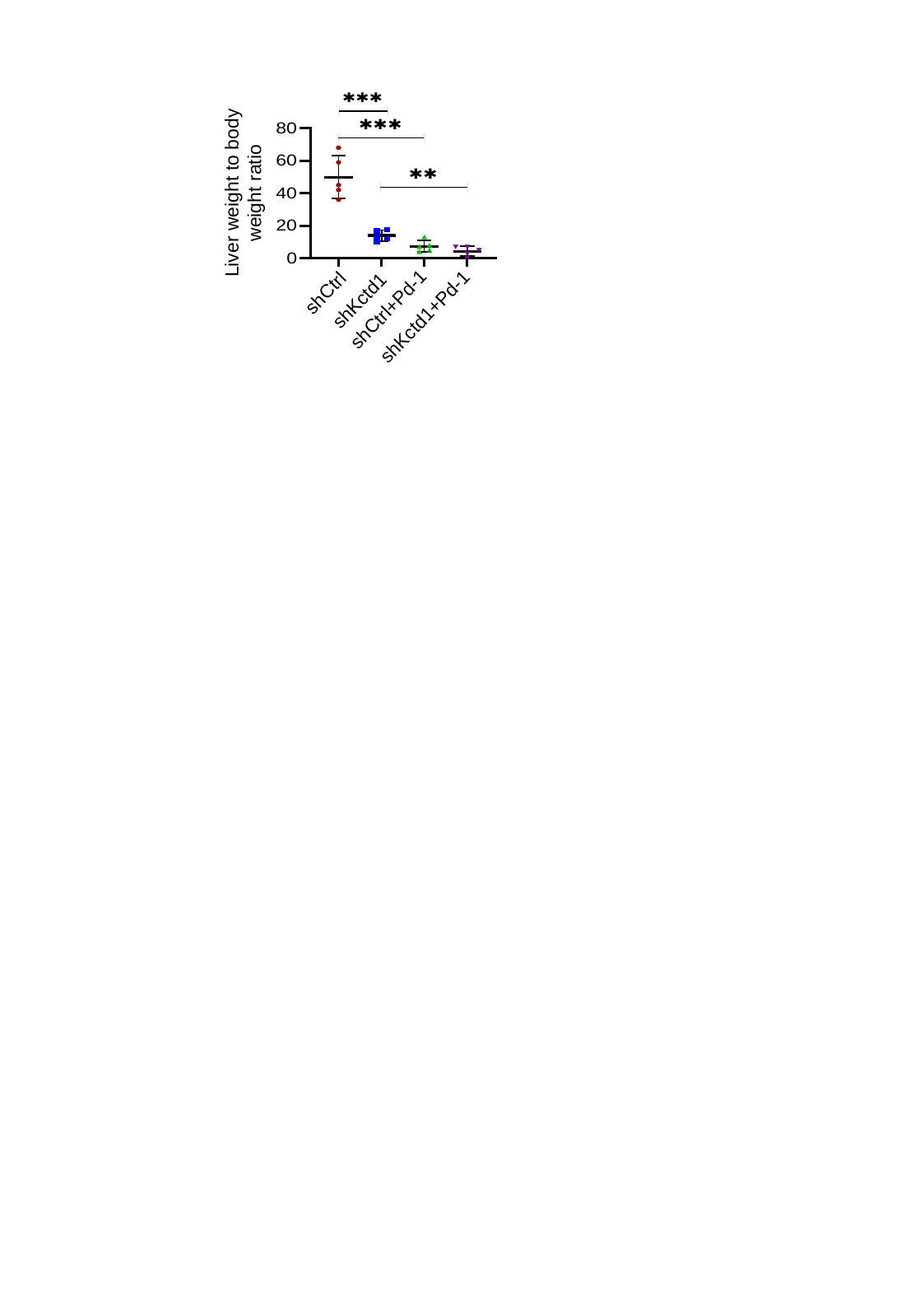

shKctd1
shCtrl+Pd-1
shCtrl
shKctd1+Pd-1
Liver weight to body weight ratio
