# Supplementary material for: KCTD1 stabilizes c-Myc to upregulate PD-L1 and suppress anti-tumor immunity in hepatocellular carcinoma
Source: Cell Death Discov. 2026 Mar 2;12:129. doi: 10.1038/s41420-026-02975-6 (PMC13039683; doi:10.1038/s41420-026-02975-6)
Supplement: Supplementary file 2 — supplemental information [file 41420_2026_2975_MOESM2_ESM.docx]

**Supporting information**

**KCTD1 Stabilizes c-Myc to Upregulate PD-L1 and Suppress Anti-Tumor Immunity in Hepatocellular Carcinoma**

Dongmei Zhong^1,2^, Shengwen Long^1^, Yilan Dai^1^, Yaru Yin^1,2^, Zixin Zhang^1,2^, Mi Ouyang^1,2^, Xinyu Zhu^1^, Anyi Hou^1^, Yanling Qin^1^, Qinghao Wang^1,2^, Mengting Gong^3,1†^, Xiaofeng Ding^1,2†^

1 The National & Local Joint Engineering Laboratory of Animal Peptide Drug Development, College of Life Science, Hunan Normal University, Changsha, 410081, China

2 Institute of Interdisciplinary Studies, Hunan Normal University, Changsha, 410081, China

3 College of Physical Education, Hunan University of Finance and Economics, Changsha, 410205, China

† Correspondence: Mengting Gong, Ph. D., [gmt20130219@hotmail.com](mailto:gmt20130219@hotmail.com); Xiaofeng Ding, Ph. D., [dingxiaofeng@hunnu.edu.cn](mailto:dingxiaofeng@hunnu.edu.cn)

Subtitle: Targeting the KCTD1–c-Myc axis improves anti–PD-1 immunotherapy efficacy

**SUPPLEMENTAL TABLES**

**Table S1. Primer pairs used in this study.**

|  | Sequence（5’-3’） | Purpose |
| --- | --- | --- |
| c-Myc 1 F | GGAATTCCGAACGTTAGCTTCACCAACAG | PCR |
| c-Myc 1 R | GGGGTACCTTACATACAGTCCTGGATGATGA | PCR |
| c-Myc 2 F | GGAATTCCGTTGTACCTGCAGGATCTGAG | PCR |
| c-Myc 2 R | GGGGTACCTTAACTCTGACACTGTCCAACTT | PCR |
| c-Myc 3 F | GGAATTCCGAGACAGATCAGCAACAACCG | PCR |
| c-Myc 3 R | GGGGTACCTTACGCACAAGAGTTCCGTA | PCR |

**Table S2. Weights (g) of the main organs after the treatment with anti-Pd1.**

|  | shCtrl | shKCTD1 | Anti-Pd-1 | |
| --- | --- | --- | --- | --- |
|  |  |  | shCtrl | shKCTD1 |
| Body(g) | 15.48  14.13  14.65  14.56  14.97 | 18.54  16.85  17.23  16.37  16.25 | 17.93  16.38  17.02  15.42  15.01 | 15.74  17.70  14.42  16.24  13.46 |
| Spleen(g) | 0.1025  0.0745  0.0880  0.0828  0.1196 | 0.0851  0.0890  0.0791  0.0762  0.1066 | 0.1085  0.0817  0.0764  0.0744  0.0828 | 0.0901  0.0750  0.0876  0.0673  0.0929 |
| Liver(g) | 2.550  1.858  1.501  1.731  2.062 | 1.395  1.314  1.211  1.236  1.309 | 1.198  1.145  1.101  1.169  0.953 | 0.729  0.924  0.989  0.854  0.802 |

**SUPPLEMENTAL FIGURE LEGENDS**

**Fig. S1: Silver staining of KCTD1-interacting proteins in MHCC97H cells.**

co-IP was performed using anti-KCTD1 antibodies in MHCC97H cells. The precipitated complexes were separated by SDS-PAGE and visualized by silver staining to detect KCTD1-interacting proteins.

**Fig. S2: Western blot analysis of PD-1 expression in CD8^+^ T cellsfrom the co-culture system of CD8⁺ T cells and MHCC97H tumor cells.**

**Fig. S3: Detection of Kctd1 shRNA lentiviral infection efficiency in Hepa1-6 Cells.**

Fluorescence imaging was used to confirm shRNA infection efficiency and Kctd1 knockdown in Hepa1-6 cells by detecting GFP fluorescence.

**Fig. S4: Effect of CD8⁺ T cell depletion on intrahepatic tumor growth.
A** Experimental strategy for CD8α blockade treatment of intrahepatic HCC in C57BL/6 mice. **B, C, D** Representative liver images, quantification of liver tumor nodules, and liver-to-body weight ratio in mice treated with Kctd1-knockdown cells and/or anti-CD8 antibodies. **E,F** IHC analysis showing CD8⁺ T cell depletion in tumor tissues and quantification of CD8-positive cells.

**Fig. S5: Quantification of liver to body weight ratio in mice treated with Kctd1-knockdown cells and/or PD-1 antibodies.**
