# Supplementary figures and images for: KCTD1 stabilizes c-Myc to upregulate PD-L1 and suppress anti-tumor immunity in hepatocellular carcinoma
Source: Cell Death Discov. 2026 Mar 2;12:129. doi: 10.1038/s41420-026-02975-6 (PMC13039683; doi:10.1038/s41420-026-02975-6)

Fig1 F

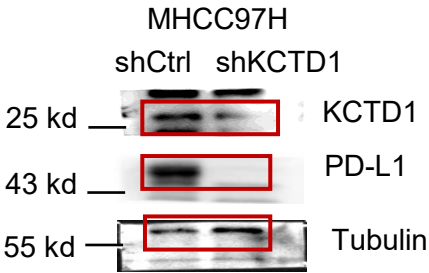

Fig1 H

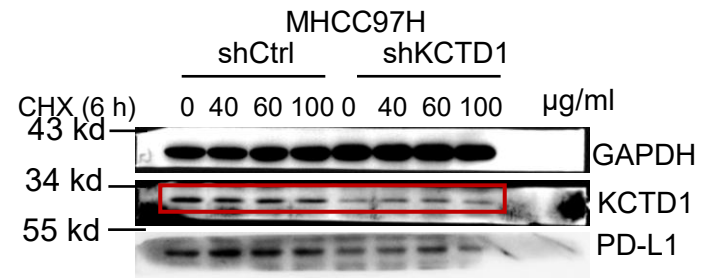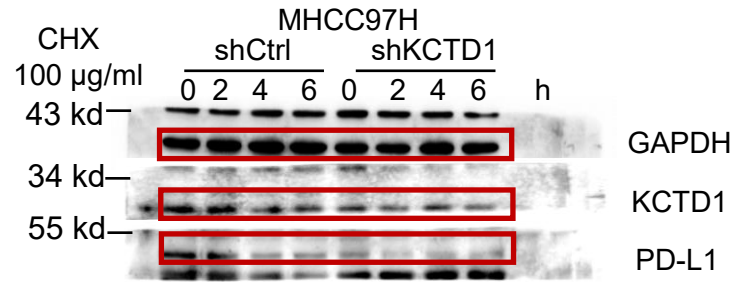

Fig1 I

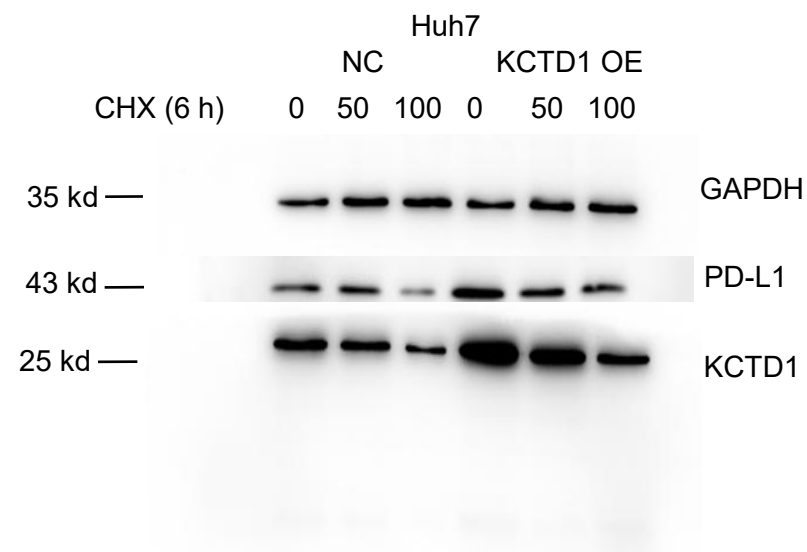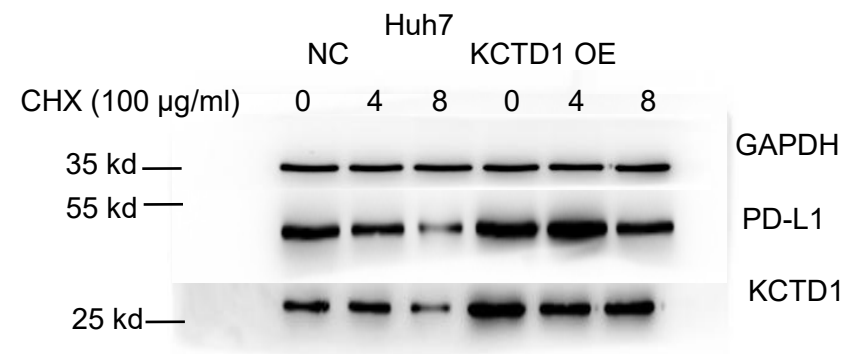

Fig2 E

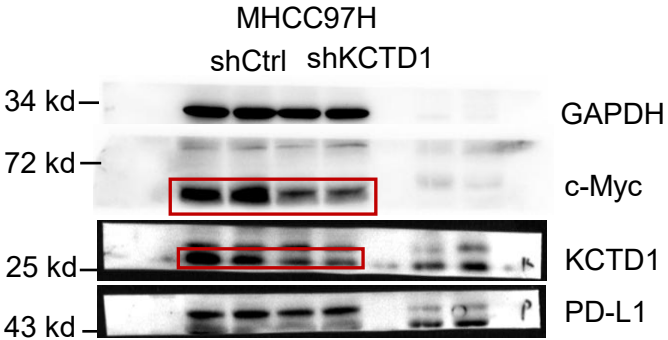

Fig2 F

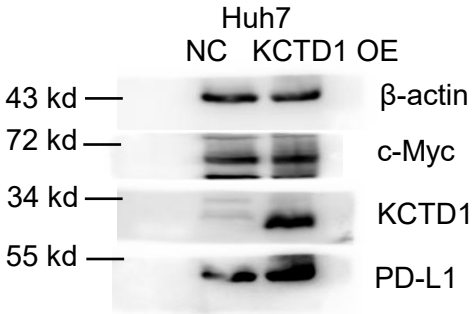

Fig2 G

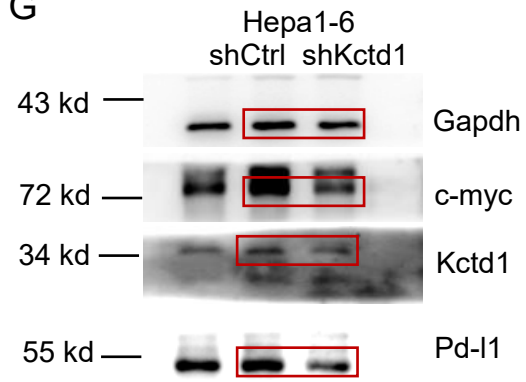

Fig 2 H

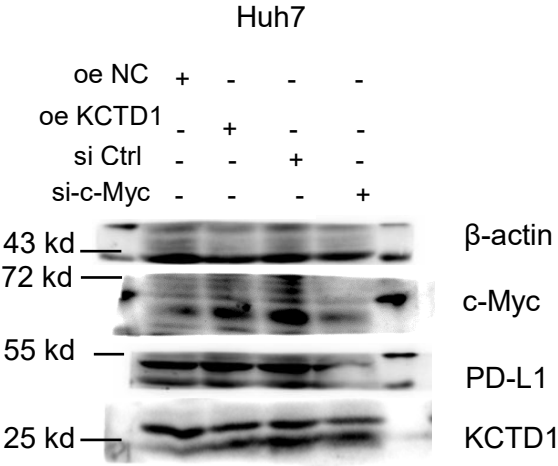

Fig2 I

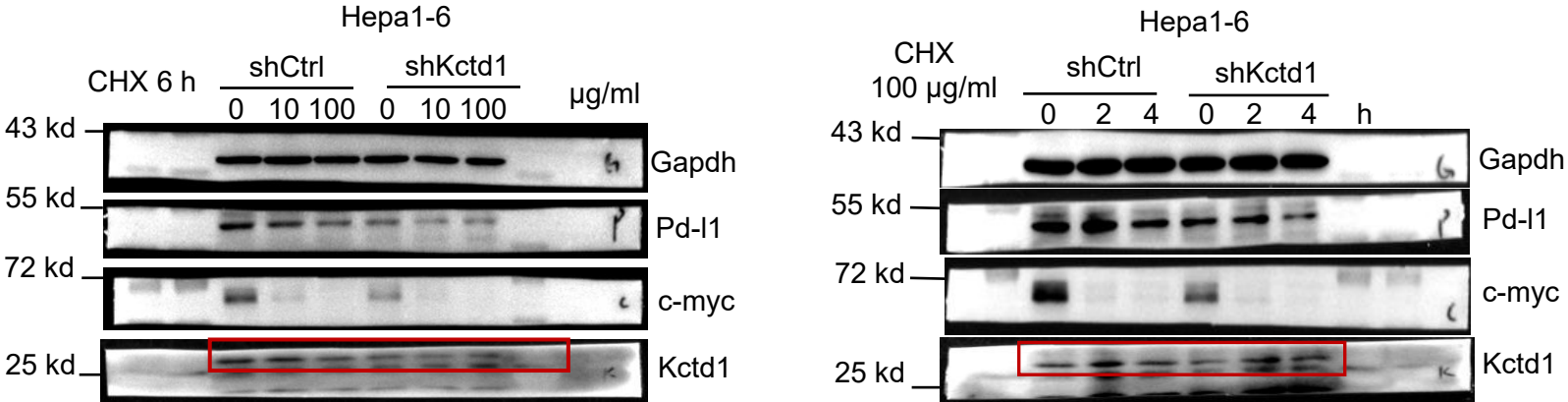

Fig3 B

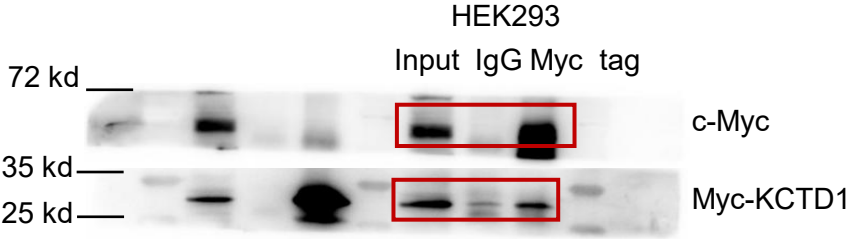

Fig3 C

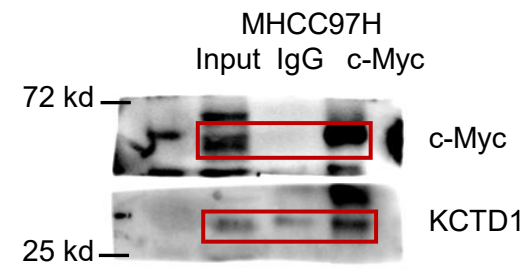

Fig3 E

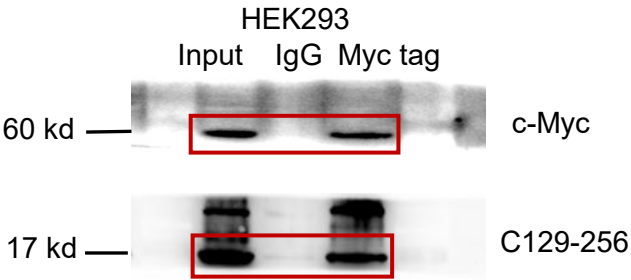

Fig3 F

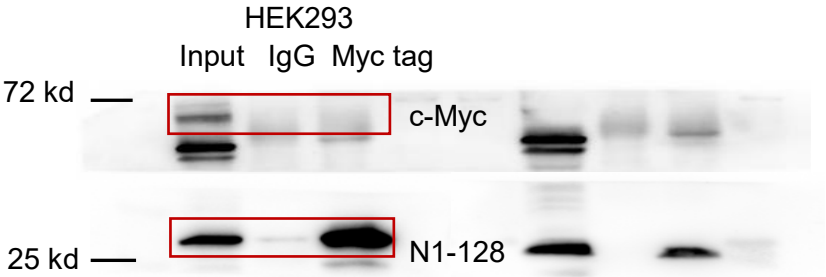

Fig3 G

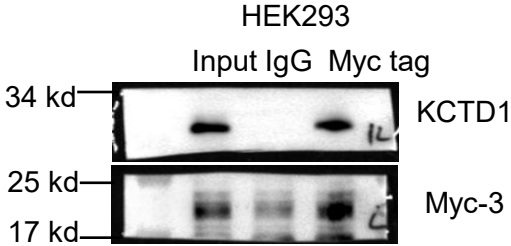

Fig3 H

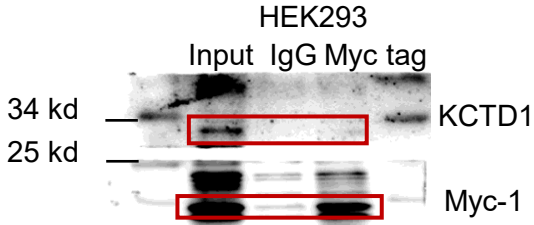

Fig3 I

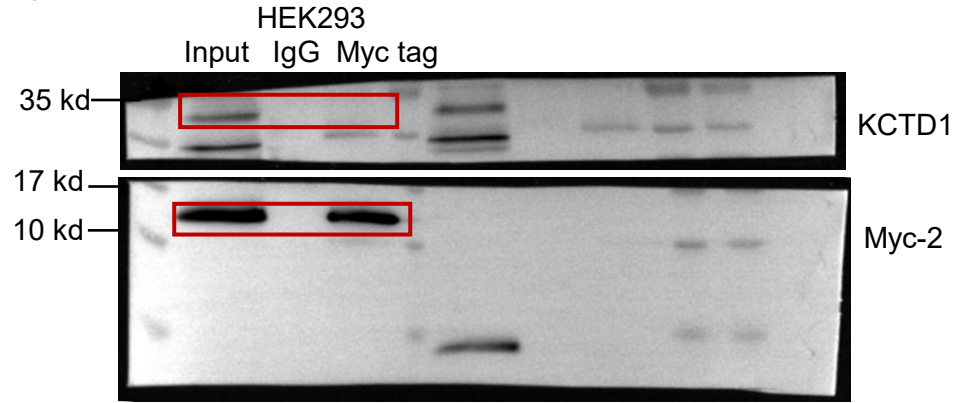

Fig 4J

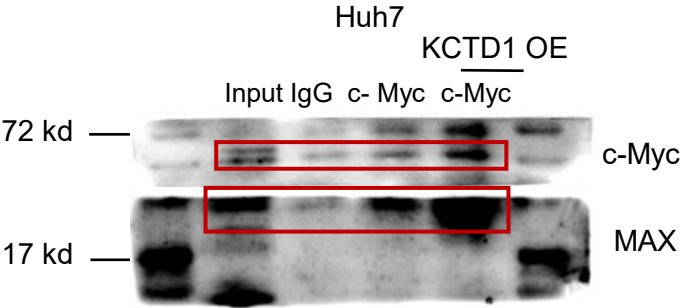

Fig5 A

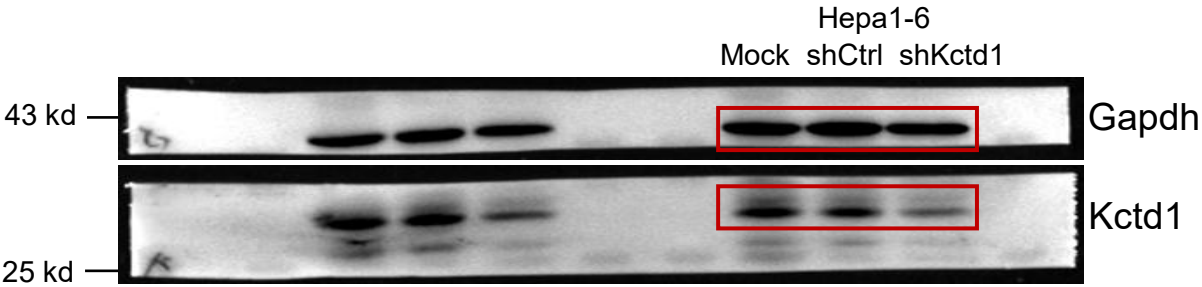

Supplement: Supplementary file 3 — original uncropped [file 41420_2026_2975_MOESM3_ESM.pdf]
